# Supplementary material for: Inhibition of p38-MK2 pathway enhances the efficacy of microtubule inhibitors in breast cancer cells
Source: eLife. 2025 May 29;13:RP104859. doi: 10.7554/eLife.104859 (PMC12122001; doi:10.7554/eLife.104859)
Supplement: MDAR checklist [file elife-104859-mdarchecklist1.docx]

**Materials Design Analysis Reporting (MDAR)**

**Checklist for Authors**

The [MDAR framework](https://osf.io/xfpn4/) establishes a minimum set of requirements in transparent reporting mainly applicable to studies in the life sciences.

*eLife* asks authors to **provide detailed information within their article** to facilitate the interpretation and replication of their work. Authors can also upload supporting materials to comply with relevant reporting guidelines for health-related research (see [EQUATOR Network](http://www.equator-network.org/%20)), life science research (see the [BioSharing Information Resource](http://biosharing.org/)), or animal research (see the [ARRIVE Guidelines](http://www.plosbiology.org/article/info:doi/10.1371/journal.pbio.1000412) and the [STRANGE Framework](https://doi.org/10.1038/d41586-020-01751-5); for details, see *eLife*’s [Journal Policies](https://reviewer.elifesciences.org/author-guide/journal-policies)). Where applicable, authors should refer to any relevant reporting standards materials in this form.

For all that apply, please note **where in the article** the information is provided. Please note that we also collect information about data availability and ethics in the submission form.

**Materials:**

| **Newly created materials** | **Indicate where provided: section/figure legend** | **N/A** |
| --- | --- | --- |
| The manuscript includes a dedicated "materials availability statement" providing transparent disclosure about availability of newly created materials including details on how materials can be accessed and describing any restrictions on access. |  | NA |
|  |  |  |
| **Antibodies** | **Indicate where provided: section/figure legend** | **N/A** |
| For commercial reagents, provide supplier name, catalogue number and [RRID](https://scicrunch.org/resources), if available. | anti-Phospho-HSP27 (Ser82) (Rabbit monoclonal), Cell Signaling Technology, Cat# 9709S, RRID: AB_11217429. /Figure 6-Supplement 1A and Methods. |  |
|  |  |  |
| **DNA and RNA sequences** | **Indicate where provided: section/figure legend** | **N/A** |
| Short novel DNA or RNA including primers, probes: Sequences should be included or deposited in a public repository. |  | NA |
|  |  |  |
| **Cell materials** | **Indicate where provided: section/figure legend** | **N/A** |
| Cell lines: Provide species information, strain. Provide accession number in repository OR supplier name, catalog number, clone number, OR RRID. | • hTERT RPE-1 (*Homo-sapiens*), ATCC, CRL-4000  • MDA-MB-231 (*Homo-sapiens*), ATCC, CRM-HTB-26  • CAL-51 (*Homo-sapiens*), RRID:CVCL_1110  • T-47D (*Homo-sapiens*), ATCC, HTB-133  • MCF10A (*Homo-sapiens*), ATCC, CRL-10317  • p53-KO CAL-51 (*Homo-sapiens*), Redman-Rivera *et al.*, Nat Commun. (2021)  • Histone H2B-mScarlet, α-tubulin-mNeonGreen CAL-51 (*Homo-sapiens*), Scribano *et al.*, Sci Transl Med. (2021)  / Methods. |  |
| Primary cultures: Provide species, strain, sex of origin, genetic modification status. |  | NA |
|  |  |  |
| **Experimental animals** | **Indicate where provided: section/figure legend** | **N/A** |
| Laboratory animals or Model organisms: Provide species, strain, sex, age, genetic modification status. Provide accession number in repository OR supplier name, catalog number, clone number, OR RRID. | **Laboratory Animals and Ethics Approval**   - **Species:** Mouse - **Strain:** NOD/ShiJic-scidJcl - **Sex:** Female - **Age:** 7 weeks - **Genetic modification:** None - **Supplier:** CLEA Japan, Inc. |  |
| Animal observed in or captured from the field: Provide species, sex, and age where possible. |  | NA |
|  |  |  |
| **Plants and microbes** | **Indicate where provided: section/figure legend** | **N/A** |
| Plants: provide species and strain, ecotype and cultivar where relevant, unique accession number if available, and source (including location for collected wild specimens). |  | NA |
| Microbes: provide species and strain, unique accession number if available, and source. |  | NA |
|  |  |  |
| **Human research participants** | **Indicate where provided: section/figure legend) or state if these demographics were not collected** | **N/A** |
| If collected and within the bounds of privacy constraints report on age, sex, gender and ethnicity for all study participants. |  | **NA** |

**Design:**

| **Study protocol** | **Indicate where provided: section/figure legend** | **N/A** |
| --- | --- | --- |
| If the study protocol has been pre-registered, provide DOI. For clinical trials, provide the trial registration number OR cite DOI. |  | NA |
|  |  |  |
| **Laboratory protocol** | **Indicate where provided: section/figure legend** | **N/A** |
| Provide DOI OR other citation details if detailed step-by-step protocols are available. | Our Methods include protocols and citations. |  |
|  |  |  |
| **Experimental study design (statistics details) *** | | |
| **For in vivo studies: State whether and how the following have been done** | **Indicate where provided: section/figure legend. If it could have been done, but was not, write “not done”** | **N/A** |
| Sample size determination | Sample size calculation was performed assuming a baseline improvement rate of 40% with the existing treatment (PTX) and an expected improvement of 10% (to 50%) for the new treatment (CMPD1), using a one-tailed test with α = 0.05 and β = 0.20 (80% statistical power). Based on these parameters, we determined that 11 mice per group were required, and thus assigned this number of animals to each of the three experimental groups: no-treatment control, PTX, and CMPD1. |  |
| Randomisation | Mice were randomly assigned to each treatment group and allocated unique identification numbers, and ensuring balanced tumor volumes at baseline |  |
| Blinding | All measurements of tumor diameter and body weight were conducted in a blinded manner, with the investigator unaware of the group assignments. Furthermore, surgical procedures and subsequent analyses were performed without knowledge of the treatment groups, and group identities were disclosed only during the final data analysis. |  |
| Inclusion/exclusion criteria | All mice were carefully monitored for health status throughout the experimental period. Only healthy mice, exhibiting normal behavior, stable body weight, and no signs of infection or physical abnormalities were included. Mice showing any deterioration of general condition, significant weight loss (>20%), severe tumor ulceration, or signs of distress were promptly excluded from the study, in accordance with institutional animal welfare guidelines. |  |
|  |  |  |
| **Sample definition and in-laboratory replication** | **Indicate where provided: section/figure legend** | **N/A** |
| State number of times the experiment was replicated in the laboratory. | All information included in Methods or Legends. All experiments performed at least 2 biological replicates. |  |
| Define whether data describe technical or biological replicates. | All biological replicates and mentioned in Methods or Lengeds. |  |
|  |  |  |
| **Ethics** | **Indicate where provided: section/submission form** | **N/A** |
| Studies involving human participants: State details of authority granting ethics approval (IRB or equivalent committee(s), provide reference number for approval. |  | NA |
| Studies involving experimental animals: State details of authority granting ethics approval (IRB or equivalent committee(s), provide reference number for approval. | All animal experiments were approved by the Animal Care and Use Committee in Chiba University, Japan (Dou30-423) |  |
| Studies involving specimen and field samples: State if relevant permits obtained, provide details of authority approving study; if none were required, explain why. |  | NA |
|  |  |  |
| **Dual Use Research of Concern (DURC)** | **Indicate where provided: section/submission form** | **N/A** |
| If study is subject to dual use research of concern regulations, state the authority granting approval and reference number for the regulatory approval. |  | NA |

**Analysis:**

| **Attrition** | **Indicate where provided: section/figure legend** | **N/A** |
| --- | --- | --- |
| Describe whether exclusion criteria were pre-established. Report if sample or data points were omitted from analysis. If yes, report if this was due to attrition or intentional exclusion and provide justification. |  | NA |
|  |  |  |
| **Statistics** | **Indicate where provided: section/figure legend** | **N/A** |
| Describe statistical tests used and justify choice of tests. | Details are in Methods or Legends |  |
|  |  |  |
| **Data availability** | **Indicate where provided: section/submission form** | **N/A** |
| For newly created and reused datasets, the manuscript includes a data availability statement that provides details for access (or notes restrictions on access). | Yes (no restrictions) |  |
| When newly created datasets are publicly available, provide accession number in repository OR DOI and licensing details where available. | RNA-seq data generated in this study are available at Gene Expression Omnibus under Accession Number: GSE224462:  Go to <https://www.ncbi.nlm.nih.gov/geo/query/acc.cgi?acc=GSE224462>  Additional source data are available at Dryad (DOI: 10.5061/dryad.m37pvmdf1). |  |
| If reused data is publicly available provide accession number in repository OR DOI, OR URL, OR citation. | RNA-seq data generated in this study are available at Gene Expression Omnibus under Accession Number: GSE224462:  Go to <https://www.ncbi.nlm.nih.gov/geo/query/acc.cgi?acc=GSE224462>  Additional source data are available at Dryad (DOI: 10.5061/dryad.m37pvmdf1). |  |
|  |  |  |
| **Code availability** | **Indicate where provided: section/figure legend** | **N/A** |
| For any computer code/software/mathematical algorithms essential for replicating the main findings of the study, whether newly generated or re-used, the manuscript includes a data availability statement that provides details for access or notes restrictions. |  | NA |
| Where newly generated code is publicly available, provide accession number in repository, OR DOI OR URL and licensing details where available. State any restrictions on code availability or accessibility. |  | NA |
| If reused code is publicly available provide accession number in repository OR DOI OR URL, OR citation. |  | NA |

**Reporting:**

The MDAR framework recommends adoption of discipline-specific guidelines, established and endorsed through community initiatives.

| **Adherence to community standards** | **Indicate where provided: section/figure legend** | **N/A** |
| --- | --- | --- |
| State if relevant guidelines (e.g., ICMJE, MIBBI, ARRIVE, STRANGE) have been followed, and whether a checklist (e.g., CONSORT, PRISMA, ARRIVE) is provided with the manuscript. |  | NA |

* We provide the following guidance regarding transparent reporting and statistics; we also refer authors to [Ten common statistical mistakes to watch out for when writing or reviewing a manuscript](https://doi.org/10.7554/eLife.48175).

**Sample-size estimation**

- You should state whether an appropriate sample size was computed when the study was being designed
- You should state the statistical method of sample size computation and any required assumptions
- If no explicit power analysis was used, you should describe how you decided what sample (replicate) size (number) to use

**Replicates**

- You should report how often each experiment was performed
- You should include a definition of biological versus technical replication
- The data obtained should be provided and sufficient information should be provided to indicate the number of independent biological and/or technical replicates
- If you encountered any outliers, you should describe how these were handled
- Criteria for exclusion/inclusion of data should be clearly stated
- High-throughput sequence data should be uploaded before submission, with a private link for reviewers provided (these are available from both GEO and ArrayExpress)

**Statistical reporting**

- Statistical analysis methods should be described and justified
- Raw data should be presented in figures whenever informative to do so (typically when N per group is less than 10)
- For each experiment, you should identify the statistical tests used, exact values of N, definitions of center, methods of multiple test correction, and dispersion and precision measures (e.g., mean, median, SD, SEM, confidence intervals; and, for the major substantive results, a measure of effect size (e.g., Pearson's r, Cohen's d)
- Report exact p-values wherever possible alongside the summary statistics and 95% confidence intervals. These should be reported for all key questions and not only when the p-value is less than 0.05.

**Group allocation**

- Indicate how samples were allocated into experimental groups (in the case of clinical studies, please specify allocation to treatment method); if randomization was used, please also state if restricted randomization was applied
- Indicate if masking was used during group allocation, data collection and/or data analysis
